# Supplementary material for: Ran-GTP assembles a specialized spindle structure for accurate chromosome segregation in medaka early embryos
Source: Nat Commun. 2024 Feb 1;15:981. doi: 10.1038/s41467-024-45251-w (PMC10834446; doi:10.1038/s41467-024-45251-w)
Supplement: Supplementary file 1 — Supplementary Information [file 41467_2024_45251_MOESM1_ESM.pdf]

Supplementary Information

Supplementary Discussion

Comet-like structure under oil droplets

Unexpectedly, we found that EGFP-α-tubulin accumulated under oil droplets and formed comet-like structures during oil-droplet displacement before the first mitosis (Fig. 1b). The comets contained string-like signals, suggesting that MTs are polymerized and are pushing the oil droplets (Supplementary Fig. 1d).

Centrosome positioning

In early embryonic divisions, centrosomes are always located at both sides of the nucleus before NEBD (Fig. 2d). This centrosome positioning would be critical to achieve rapid bipolar spindle formation, like a prophase pathway in somatic cells[1]. We found that there are two types of nucleus-centrosome association after mitotic exit in medaka early embryos (Fig. 2d-g). Type II is somehow more frequent in the 1st mitosis, but it seems to gradually become 50% in the 4th divisions. Intriguingly, ~23% of naturally fertilized embryos showed asymmetrical cellular patterning at the 4-cell stage[2]. The error of nucleus-centrosome interaction in the Type II pathway may be related to spindle mis-orientation and asymmetrical cellular patterning. However, these embryos developed normally and hatched on time around day 7 post-fertilization[2]. It will be interesting to determine what level of cellular asymmetry is tolerated during early embryogenesis.

Checkpoint functions in medaka early embryonic divisions

In contrast to mammals, early embryonic divisions in medaka are fast and accurate (Fig. 4a, Supplementary Fig. 4a-c) despite the lack of a functional spindle assembly checkpoint (SAC) (Fig. 5h-j). Other cell-cycle checkpoints such as the S-phase checkpoint are also non-functional in medaka early embryos[3], which increases defects of cell division or other events and causes extremely abnormal blastomeres such as enucleated cells (Fig. 6f). SAC seems to become functional at least in the blastula stage, considering that a mitotic delay was observed in RCC1-depleted cells (Fig. 9g). Several models have been proposed regarding SAC acquisition in early embryos[4]. Understanding how embryos acquire the SAC and other cell-cycle checkpoints in medaka would be an important topic in future studies.

Centrosome separation from spindle poles

In contrast to typical somatic spindles, centrosome-centrosome distance continuously increases during early embryonic divisions (Fig. 4d), which creates a large gap between spindle poles and centrosomes at metaphase and anaphase (Fig. 5b). Since dynein and NuMA are required to connect centrosomes to spindle poles in *Drosophila* S2 cells[5] and human Rpe1 cells[6], respectively, we speculate that these proteins are regulated differently in medaka early embryonic spindles, which reduces interaction between spindle poles and astral MTs and causes spindle pole-centrosome separation. Centrosomes move outward, probably due to MT-length-dependent cytoplasmic pulling forces[7,8]. These forces would also pull interphase nuclei outward, resulting in nuclear elongation along the centrosome-centrosome axis.

Medaka early embryonic spindles consist of subclasses of MTs with different characteristics

In live cells, EGFP-α-tubulin showed higher intensity at the spindle center than astral MTs at metaphase (Fig. 3b), and the ratio (Max value of spindle MTs/Max value of astral MTs) is >1.0 (Fig. 3f). However, after cold treatment and fixation using 4% PFA and acetone, the ratio decreased (Fig. 3f), suggesting that some populations of MTs around the spindle center are sensitive to cold treatment and fixation. Similarly, during early anaphase, EGFP-α-tubulin showed higher signals between separating chromosomes (Fig. 5a, c, t=1-2), but these signals were reduced after fixation (Supplementary Fig. 5a), suggesting that MTs between separating chromosomes recognized by EB1 (Fig. 5f) are also sensitive to fixation.

Interestingly, EMTB-3xGFP showed a unique intensity profile on spindles at both metaphase and anaphase. In contrast to α-tubulin and EB1, EMTB-3xGFP did not show clear accumulation at the spindle center at metaphase. Rather, it localized homogeneously on the metaphase spindle (Fig. 5e, Supplementary Fig. 5b). In addition, EMTB-3xGFP did not accumulate between separating chromosomes during anaphase (Fig. 5e). Given that this pattern is similar to images of immunofluorescence of anti-α-tubulin antibody in fixed embryos, EMTB-3xGFP may preferentially recognize stable MTs resistant to fixation, and cannot attach to dynamic, short, polymerizing MTs recognized by EB1. These results indicate that medaka early embryonic spindles consist of subclasses of MTs with different characteristics.

Mechanisms of chromosome movement toward spindle poles

Our quantification showed that separating chromosomes move closer to their respective spindle poles during anaphase so that the chromosome-pole distance decreases (Fig. 5b). Similarly, chromosomes move toward their spindle poles in nocodazole-treated blastomeres (Fig. 5k-l). Since our fixed and live images showed that MTs between chromosomes and spindle poles became shorter during anaphase (Fig. 5d-e), we favor the idea that k-fibers (Fig. 3j) are depolymerized during anaphase, as in typical anaphase in somatic cells. In addition, considering an increase of the pole-pole distance in nocodazole-treated anaphase spindles (Fig. 5k-l), sliding of anti-parallel MTs connected to sister k-fibers (bridging fibers) would also contribute to chromosome separation (Fig. 5m). In contrast to EMTB-3xGFP (Fig. 5e), EGFP-EB1 accumulated between separating chromosomes during anaphase (Fig. 5f). Plus-ends of bridging fibers may extend to generate anti-parallel MT regions during anaphase (Fig. 5m). In *C. elegans* meiosis, a combination of CLASP-dependent MT assembly and central spindle motor-mediated MT sliding is supposed to generate pushing forces for chromosome separation[9]. Analyzing function and precise localization of EB1, CLASP and Eg5 will be important for future studies.

Intriguingly, our quantification (Fig. 5b, 5l) showed that separating chromosomes move faster (~3-4 μm/min in controls and ~1.6-2.5 μm/min in nocodazole-treated blastomeres) than typical anaphase chromosomes (~1 μm/min)[10]. Early embryos could achieve this by increasing k-fiber depolymerization activity and/or sliding of bridging fibers, or by exerting additional mechanisms in parallel.

Transient deformed nuclear structure after mitosis

After anaphase, the chromosome mass remained near the pole (Fig. 4a t=12-14); however, it suddenly started to move toward centrosomes with a transient deformed structure in the next frame (t=15). This deformed structure was always observed from the 1st to the 4th divisions (Supplementary Fig 4a-c, n>25). We have not yet analyzed the timing of nuclear envelope reassembly after mitotic exit. However, mCh-Ol-RanQ72L, which shows nuclear membrane-like localization, accumulated on chromosome mass during chromosome movement (Supplementary Fig. 6g), suggesting that this chromosome movement occurs in concert with nuclear envelope reassembly. To understand this chromosome movement, it will be important to visualize centromere and nuclear envelope components during this process.

Nuclear size and shape regulation by Ran-GTP

Inhibition or depletion of RCC1 decreased nuclear size of medaka early embryonic blastomeres (Fig. 6d, 7i). We speculate that this is caused by defects of nucleocytoplasmic transport and lamin import, both of which are reported to regulate nuclear size in *Xenopus*[11]. In addition, nuclear laminae provide mechanical strength to nuclei[12]. RCC1-depleted nuclei cannot resist outward forces generated by centrosomes due to the reduced level of nuclear laminae, resulting in elongated nuclear shape (Fig. 6d, 7i). Such defects in nuclear size and mechanical integrity may promote asymmetrical nucleus-centrosome association (Fig. 8e), especially in the Type II pathway (Fig. 2f), leading to asymmetrical spindle formation (Fig. 6e) and unequal chromosome segregation (Fig. 6e, Fig. 8e).

Polyploid-like nuclei

We observed polyploid-like nuclei (Supplementary Fig. 2c) in embryos that later hatched. In addition, we confirmed that they can be seen in embryos that were not laser irradiated. These polyploid like nuclei were frequently observed on the surface of yolk, but not in the embryonic body, suggesting that these polyploid-like cells are generated as a natural process and have specific functions to support tissue development.

Supplementary Table 1: Plasmids established in this study.

No.	Name	Description	Related Figures
1	pTK997#8	dsDNA template having mCherry2 CDS and RCC1 homology arms	Supplementary Fig. 1a
2		pCS2+hSpCas9	Supplementary Fig. 1a, 7a
3	KI vector for EGFP-α-tubulin	pMbait-hsp-EGFP-FL-α-tubulin-BGHpA	Supplementary Fig. 1b
4	pTK1034	pCS2+EGFP-EB1	Fig. 4f, 5f
5	pTK1095	pCS2+EMTB-3xGFP	Fig. 5e, Supplementary Fig. 5b-c
6	pTK1035	pCS2+mCherry-Ol-RanT27N	Fig. 6b
7	pTK1040	pCS2+mCherry-Ol-RanWT	Fig. 6b
8	pTK1050	pCS2+OsTIR1(F74G)-P2A-mCh-H2B	Fig. 7c, d, g
9	pTK1026	pCS2+OsTIR1(F74G)-P2A-mCh-α-tubulin	Fig. 8a
10	pTK1023	dsDNA template having mAID-mClover-3xFLAG+polyA CDS and RCC1 homology arms	Supplementary Fig. 7a
11	pTK1076	pCS2+mCh-H2B	Supplementary Fig. 7b
12	pTK1073	pCS2+mCh-α-tubulin	Fig. 8a
13	pTK1063	pCS2+miRFP670nano3-H2B	Fig. 9a-b
14	pTK1097	pCS2+mCherry-Ol-RanQ72L	Fig. 6d, Supplementary Fig. 6d, g
15	pTK1030	pCS2+EGFP-H2B	Fig. 1c
16	pTK1041	pCS2+mCh-Ol-γ-tubulin	Fig. 4g

Supplementary Table 2: Medaka strains used in this study.

No.	Name	Description	Plasmids used
1	OK-Cab (MT830)	National Bio-Resource Project Medaka	
2	RCC1-mCh	A knock-in strain having mCherry at the RCC1 locus.	pTK997#8, pCS2+hSpCas9
3	EGFP-α-tubulin	A knock-in strain having EGFP-α-tubulin at the tubulin-α-1B locus	KI vector for EGFP-α-tubulin, pCS2+hSpCas9
4	RCC1-mCh + EGFP-α-tubulin	A double knock-in strain obtained by crossing No.2 with No.3 strains.	
5	RCC1-mACF	A knock-in strain having mAID-mClover-3xFLAG (mACF) at the RCC1 locus.	pTK1023, pCS2+hSpCas9

Supplementary Table 3: gRNA sequences for CRISPR/Cas9-mediated genome editing

Gene locus	gRNA (5'-3')	PAM
RCC1 (C-terminus)	GGAGGGTCGTGAAGTACTGT	TGG
Tubulin alpha-1B (N-terminus)	AAAGATGAGATAGCAAAGAC	AGG

Supplementary Table 4: PCR primers used to confirm gene editing

Gene	Primer sequence	Primer name	Figures
RCC1	AGAGTGTGAGCTGCAGTCAC	oAK35	Supplementary Fig. 1a and 7a, FW
RCC1	GTTGCTTTACTGCAGCCACC	oAK36	Supplementary Fig. 1a and 7a, RE
Tubulin alpha-1B	AGTTGCTTCTTGCTGAAGAC	oTK1290	Supplementary Fig. 1b FW
Tubulin alpha-1B	CTGGTTGGGGAAGTCCAGTC	oAK5	Supplementary Fig. 1b, RE1
Tubulin alpha-1B	ACACCTCGTCGGGGAAAAGTCCTG	oTK1291	Supplementary Fig. 1b, RE2

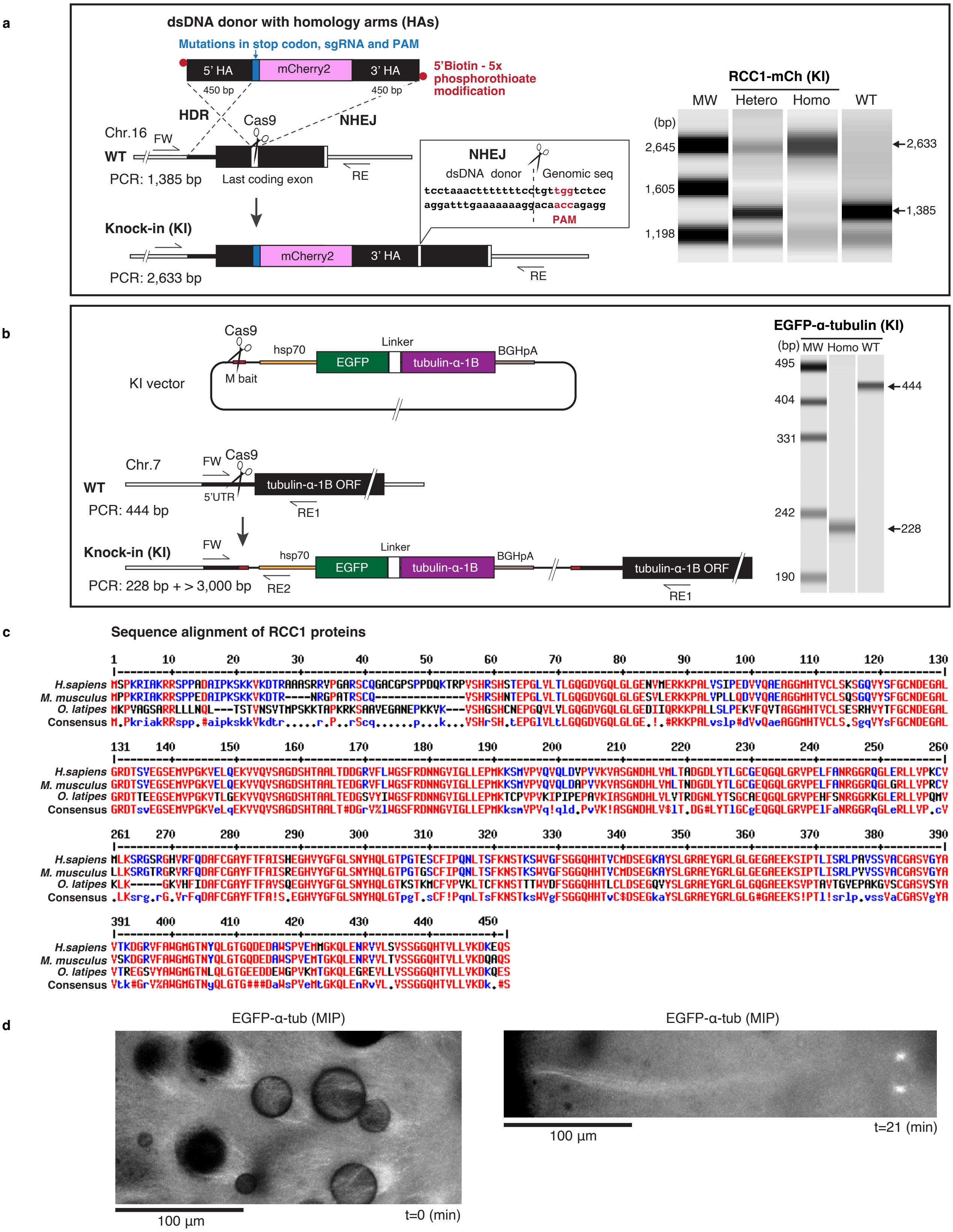

Supplementary Fig. 1. Generation of transgenic medaka strains. a Left: Schematic representation of generation of the RCC1-mCherry (RCC1-mCh) knock-in (KI) strain using dsDNA as a donor. Although HDR-mediated knock-in was expected, the genomic PCR result of the candidate strain showed a longer band (~2.6 kb). Sequence analyses of amplified fragments confirmed that the 3' homology arm (HA) of the dsDNA donor was integrated into the genome via NHEJ, but not HDR. Right: PCR-based genotyping of the RCC1 gene in the parental wild-type (WT) and KI strains. A single band of around 2.6 kb confirms homozygous insertion in the KI strain. **b** Left: Schematic representation of generation of the EGFP-α-tubulin KI strain. See Methods for details. Right: PCR-based genotyping of the EGFP-α-tubulin KI strain using three primers, FW, RE1 and RE2. A 228 bp band without a 444-bp band confirms homozygous insertion of the EGFP-α-tubulin construct. **c** Amino acid sequence alignment of RCC1 proteins in *H. sapiens* (https://www.ncbi.nlm.nih.gov/protein/NP_001041659), *M. musculus* (https://www.ncbi.nlm.nih.gov/protein/NP_001184011), and *O. latipes* (https://www.ncbi.nlm.nih.gov/protein/XP_023820025) using MultAlin (http://multalin.toulouse.inra.fr/multalin/). **d** MIP images of EGFP-α-tubulin in early (t=0, left) and late (t=21, right) stages during oil droplet displacement. Scale bars = 100 µm.

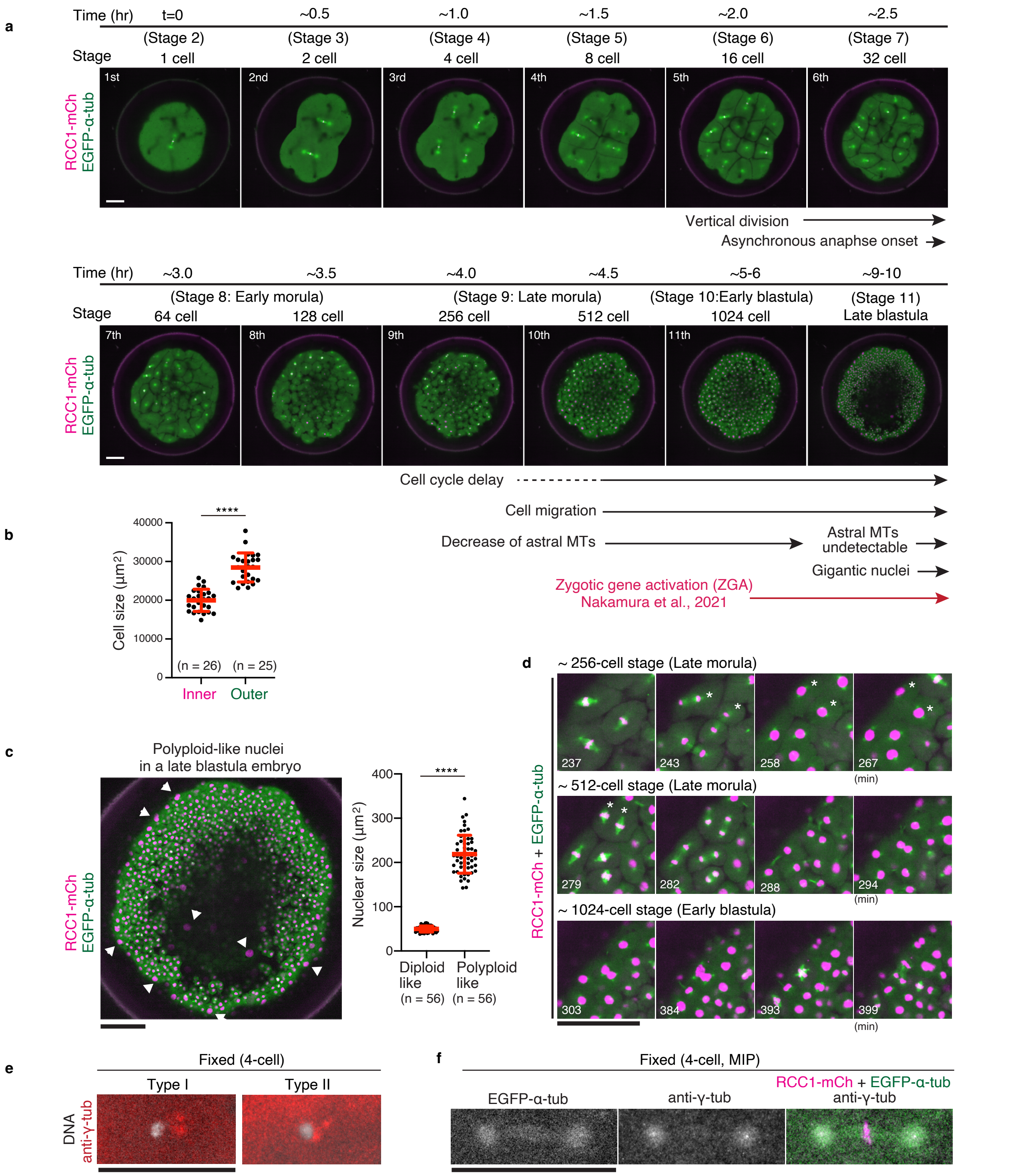

Supplementary Fig. 2. Live-cell images of early medaka embryos. a Representative live-cell images showing metaphase spindles in indicated embryonic stages. Single z-section images are shown. Vertical divisions start from the 5th division at the 16-cell stage. Structural changes of spindles roughly coincide with cell migration, cell cycle remodeling, and zygotic gene activation around early blastula stage. **b** Quantification of cell size at the 8-cell stage showing that inner blastomeres are smaller than outer blastomeres. **c** Left: A live image of a late blastula embryo showing a polyploid-like large nuclei formation (arrows) during normal development. Right: Quantification of nuclear size of diploid-like and polyploid-like blastomeres at the late blastula stage. **d** MIP images of the 3-z-sections showing two separated daughter cells (asterisks) were fused, resulting in generation of polyploid-like cells. **e** Immunofluorescence images of DNA (Hoechst 33342 staining) and γ-tubulin showing Type I and II configurations during nucleus-centrosome association after mitotic exit. Brightness was differently adjusted between these images to highlight the signals of DNA and γ-tubulin. **f** Immunofluorescence images of a fixed metaphase spindle showing signals recognized by anti-γ-tubulin antibody together with EGFP-α-tubulin and RCC1-mCherry. Error bars indicate mean ± SD. Scale bars = 100 μm. Source data for (**b-c**) are provided as a Source Data file.

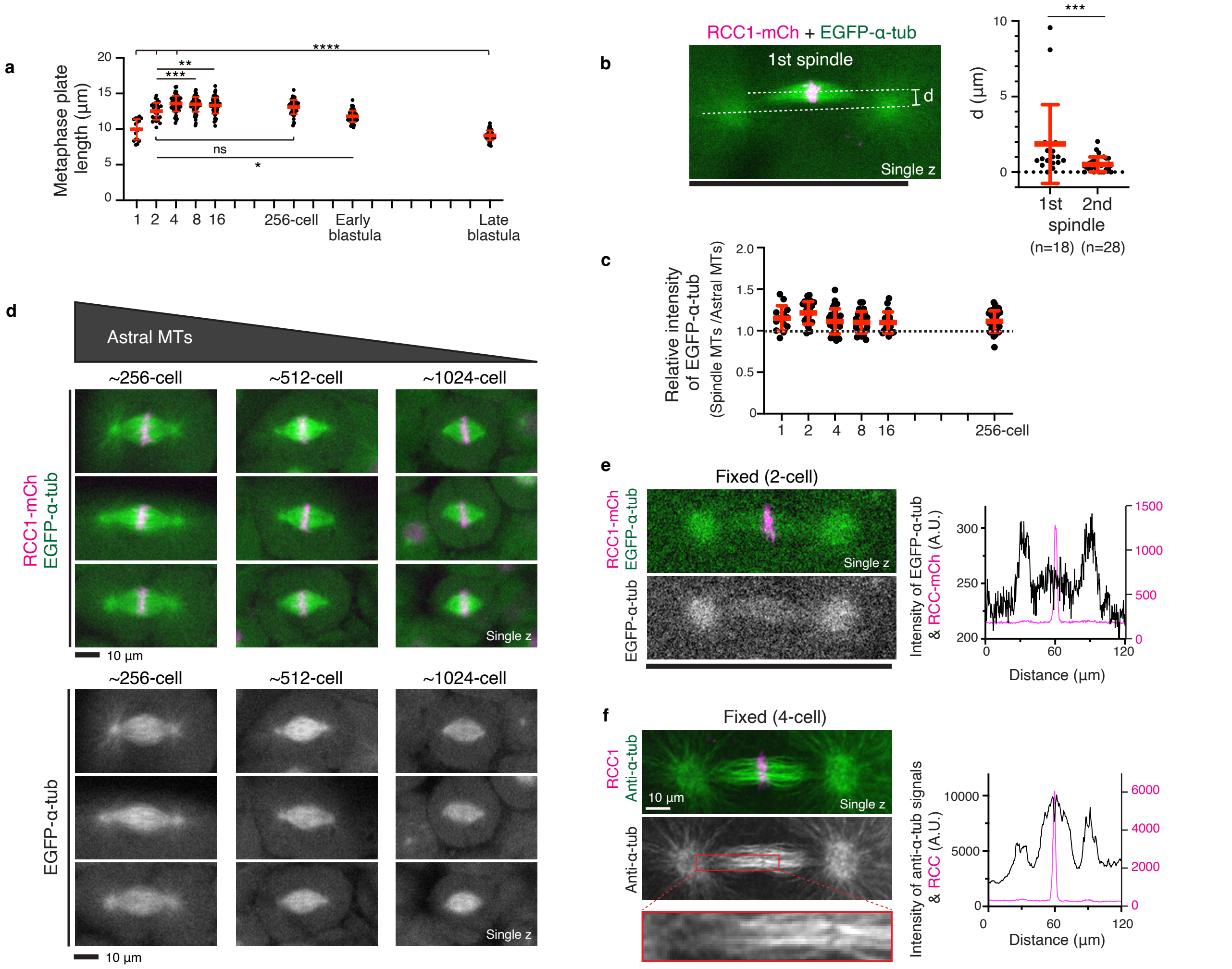

Supplementary Fig. 3. Morphological dynamics of medaka early embryonic spindles. a Quantification of metaphase plate length in indicated stages. N= 18, 29, >50 for 1-, 2-cell, and later stages, respectively, from 18 embryos. **b** Left: A live fluorescent image showing a bent zygotic spindle. Right: A graph showing distance d indicated in the left image in the 1st and 2nd spindles. The 1st spindles tend to have a large d. **c** Quantification of the max value of EGFP-α-tubulin intensity on spindle MTs relative to that on astral MTs from 1-, 2-, 4-, 8-, 16-, and 256-cell spindles. n>22 except for 1-cell (n=13). **d** Live metaphase-spindle images in ~256-, ~512-, and ~1024-cell stage embryos showing a gradual decrease of astral MTs. Single z-section images are shown. **e** Images of a metaphase spindle after fixation showing reduced fluorescence intensities of EGFP-α-tubulin at the spindle center relative to those at centrosomal regions (left). Fluorescence intensity profile of EGFP-α-tubulin on the spindle long axis (right). **f** Immunofluorescence images of metaphase spindle by anti-α-tubulin antibody. Chromosomes are visualized by RCC1-mAID-mClover-3xFLAG (left). A graph for the line scan (right) of anti-α-tubulin antibody signals. Error bars indicate mean ± SD. Scale bars = 100 µm (**b, e**) and 10 µm (**d, f**). Source data for (**a-c**, **e-f**) are provided as a Source Data file.

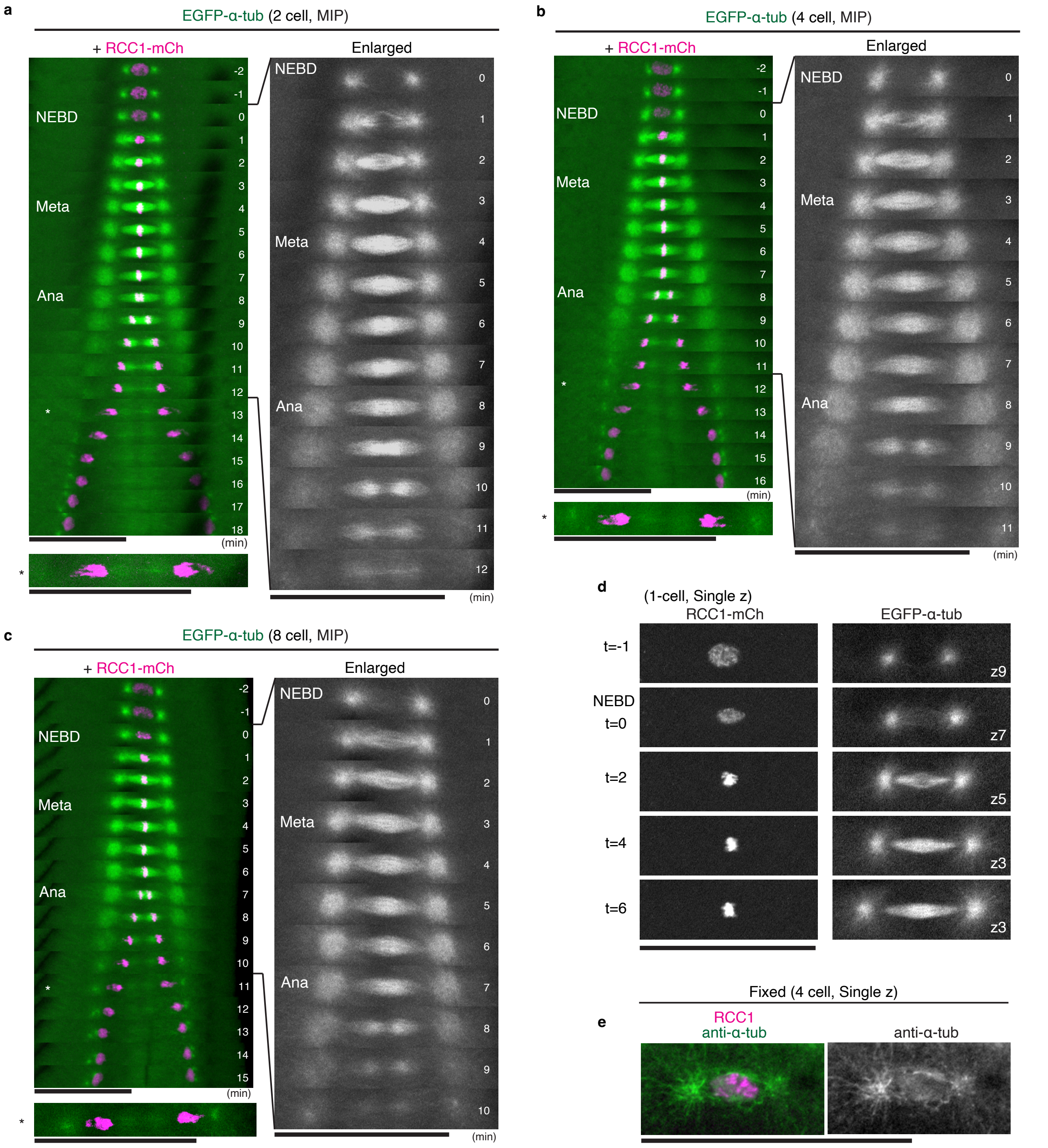

Supplementary Fig. 4. Assembly dynamics of a dense midplane MT network in early embryonic spindles. **a-c** Kymographs showing the second (**a**), third (**b**), and fourth (**c**) mitotic spindle assembly processes in a medaka embryo. A dense midplane MT network is formed during metaphase, as observed in the first mitosis (Fig. 4a). Asterisks indicate a telophase chromosome or deformed nucleus migrating to centrosomes. **d** Live-cell, single z-section images during zygotic spindle assembly. Focal z-section positions change during embryonic spindle assembly. **e** Immunofluorescence images showing MTs around the nucleus. The nucleus was visualized by RCC1-mACF. Scale bars = 100 μm.

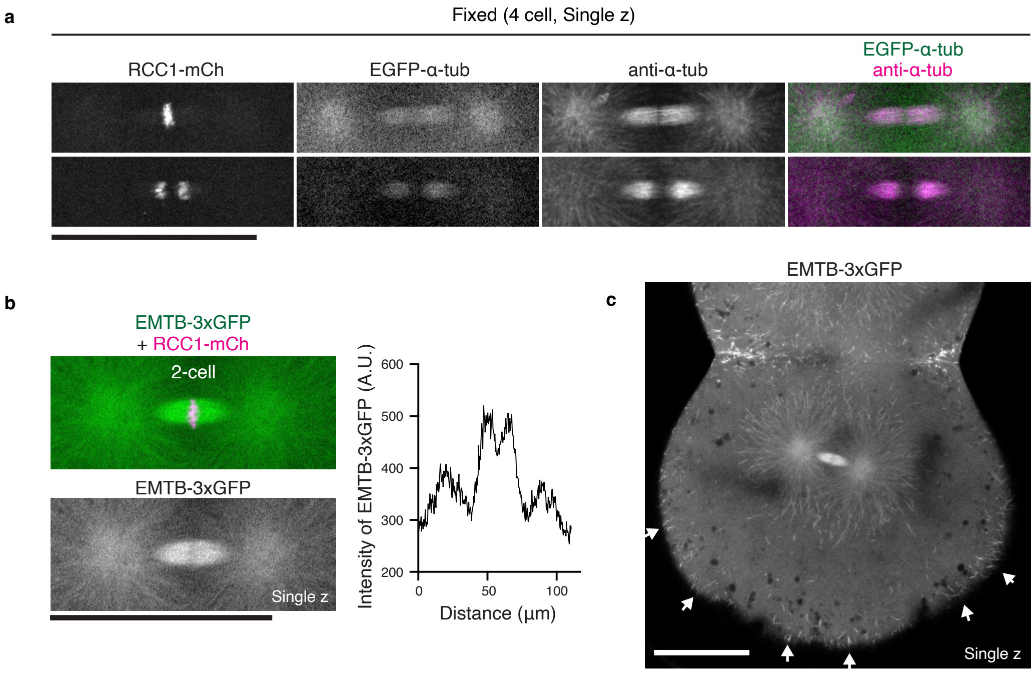

Supplementary Fig. 5. Images of spindle MTs in metaphase and anaphase. a Immunofluorescence images of fixed spindles in metaphase and anaphase using anti-α-tubulin antibody. EGFP-α-tubulin signals co-localized with signals of anti-α-tubulin antibody. However, in contrast to live cell images (Fig. 5a), EGFP-α-tubulin did not show clear accumulation at the spindle center in metaphase (top) or between separating chromosomes in anaphase (bottom), suggesting that these MTs are sensitive to our fixation protocol. **b** Live metaphase-cell images (left) and a graph for the line scan (right) of EMTB-3xGFP, showing the relative decrease of EMTB-3xGFP around the spindle midplane. **c** A single z-section image of EMTB-3xGFP showing clear astral microtubules in a 2-cell embryo. EMTB-3xGFP also displayed microtubule-like signals around cortical cell membrane (arrows), which are hardly detectable using EGFP-α-tubulin. Considering the different localization patterns between EMTB-3xGFP and EGFP-EB1 (Fig. 5f), EMTB-3xGFP may preferentially recognized relatively longer, stabilized MTs. Scale bars = 100 μm. Source data for (**b**) are provided as a Source Data file.

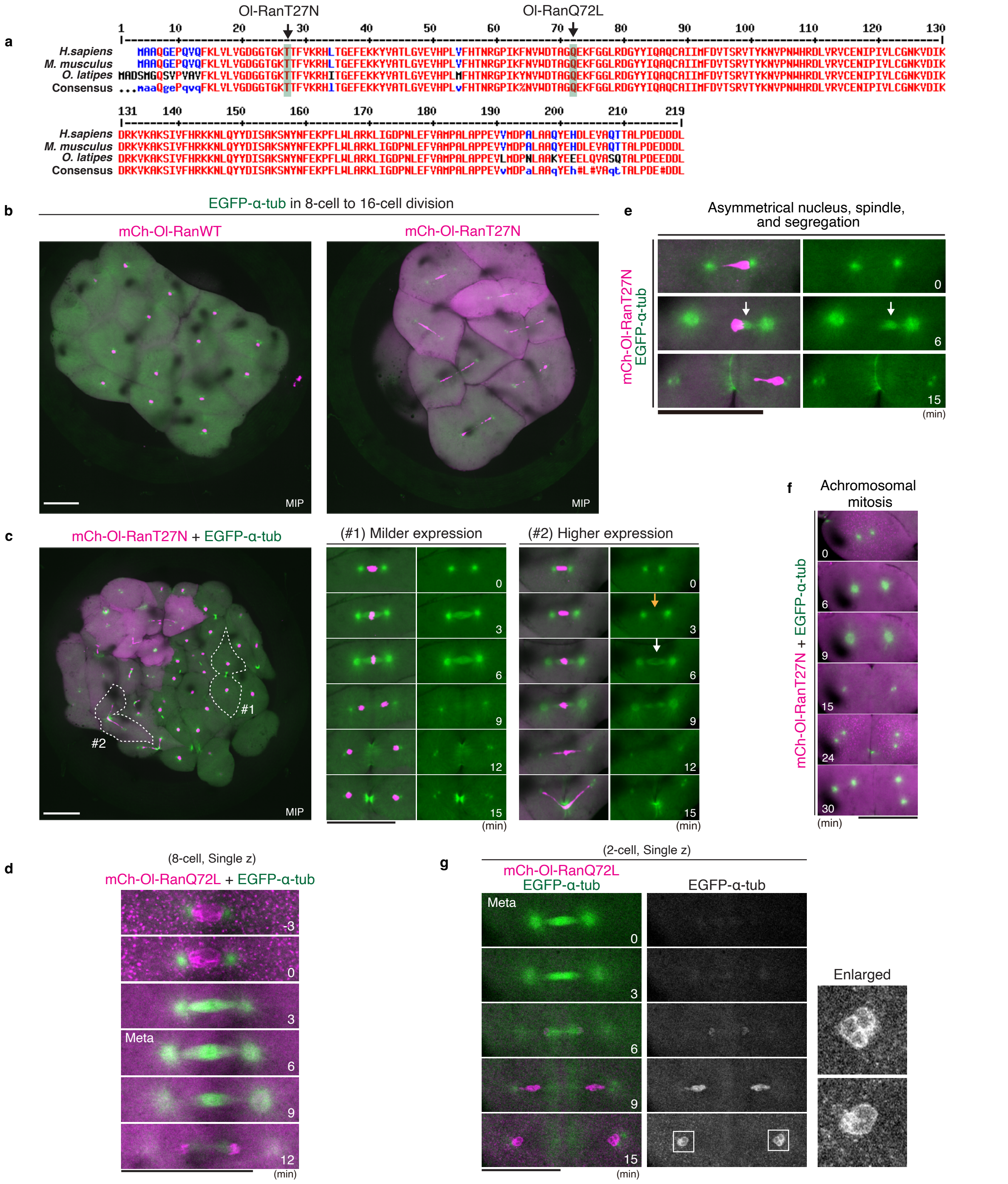

Supplementary Fig. 6. Mitotic phenotypes in RanT27N- or RanQ72L-expressing embryos. a Amino acid sequence alignment of Ran proteins in *H. sapiens* (https://www.ncbi.nlm.nih.gov/protein/NP_006316.1), *M. musculus* (https://www.ncbi.nlm.nih.gov/protein/NP_033417.1), and *O. latipes* (https://www.ncbi.nlm.nih.gov/protein/XP_004073279.1) using MultAlin. **b** Live-cell images of 8-cell-stage embryos showing normal chromosome segregation in control (left) and abnormal chromosome segregation in Ol-RanT27N-expressing embryos (right). **c** Left: a whole-embryo image showing that mCh-Ol-RanT27 causes abnormal chromosome segregation in an expression-level-dependent manner. Right: time-lapse image sequences of cells showing milder (#1) or higher (#2) expression of mCh-Ol-RanT27N. A delay of spindle formation (a yellow arrow) and a defect in specialized midplane MT network formation were observed in (#2), but not (#1), cell. **d** Live images of mCh-Ol-RanQ72L expressing embryo showing that RanQ72L appears to localize at the nuclear envelope before mitosis, but diffuses in the cytoplasm from prometaphase to anaphase (t=3, 6, 9), and localizes around chromosomes after mitosis (t=12) It also shows cytoplasmic aggregations before mitosis (t=-3, 0). Obvious mitotic defects were not observed. **e** Time-lapse image sequences showing that the nucleus has asymmetrical shape with one of the centrosomes interacting with the nucleus (t=0), resulting in asymmetrical spindle formation (t=6) followed by asymmetric segregation of all chromosomes into one daughter cell (t=15). This generated a daughter cell lacking a nucleus. **f** Live images of achromosomal mitosis showing cytokinesis without assembling the spindle between centrosomes in medaka embryos. **g** Live images of mCh-Ol-RanQ72L-expressing 2-cell embryo showing that RanQ72L appears to accumulate on chromosomes during their movement toward centrosomes (t=9). Enlarged images show the accumulation of RanQ72L on the nuclear membrane (t=15), suggesting functions of Ran-GTP at the nuclear envelope. Scale bars = 100 µm.

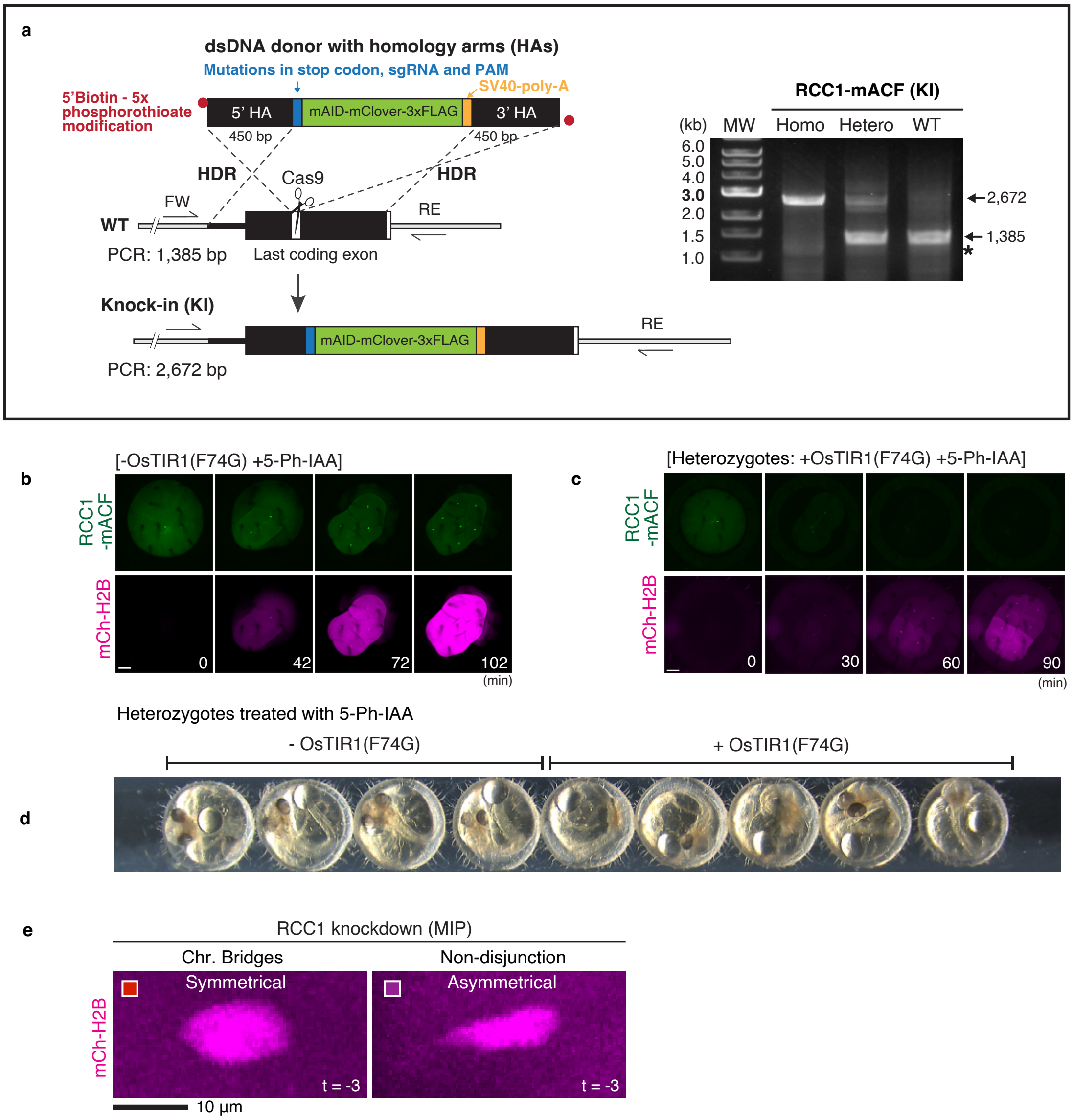

Supplementary Fig. 7. AID2-mediated depletion of RCC1 in medaka early embryos. **a** Schematic representation of generation of RCC1-mAID-mClover-3xFLAG (RCC1-mACF) knock-in (KI) strain using dsDNA as a donor. An SV40-polyA sequence is also integrated. Right: PCR-based genotyping of the RCC1 gene in the parental wild-type (WT), and homozygous and heterozygous KI strains. A single band of around 2.6 kb confirms homozygous insertion in the KI strain. An asterisk indicates a non-specific band. **b-c** Representative live-cell images showing fluorescence of RCC1-mACF and mCh-H2B in -OsTIR1(F74G) control (**b**), and heterozygous 5-Ph-IAA-treated embryos expressing OsTIR1 (F74G) (**c**). **d** Phase-contrast images of heterozygous RCC1-mACF embryos 4 days after mRNA injection and treatment with 5-Ph-IAA showing no developmental defects, regardless of the presence or absence of OsTIR1(F74G). **e** Enlarged images of nucleus in RCC1-depleted blastomeres shown in Fig. 7g. A RCC1-KD blastomere showing a chromosome non-disjunction phenotype has an asymmetrical nuclear shape (right), whereas a RCC1-depleted blastomere causing chromosome bridge displays symmetrical nuclear shape (left). Fluorescence intensities of mCh-H2B were differently adjusted between these images to highlight the shape. Scale bars = 100 µm (**b, c**) and 10 µm (**e**)

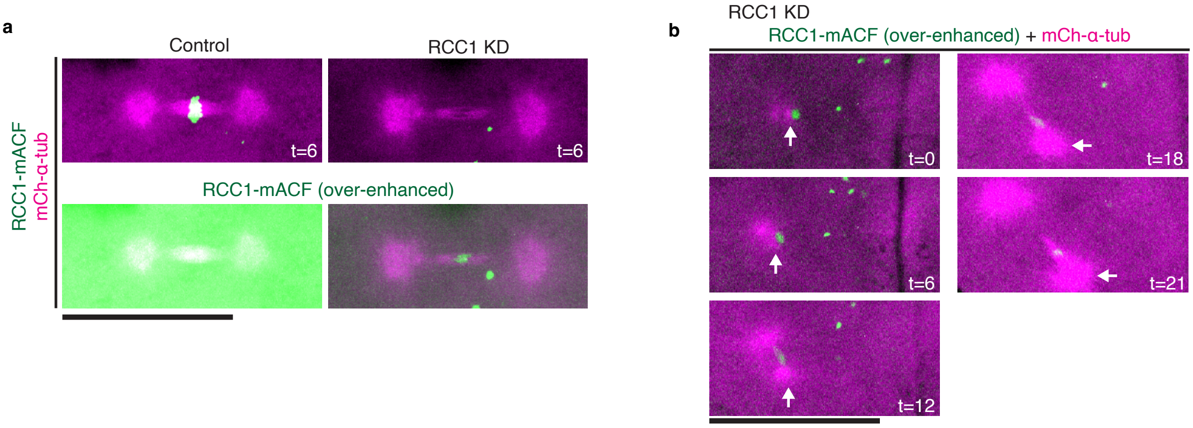

Supplementary Fig. 8. Abnormal spindles caused by AID2-mediated RCC1 depletion. a Top: Live-cell images of control (left) and RCC1 protein-knockdown (right) 4-cell blastomeres showing disruption of the dense MT network at the spindle midplane in RCC1-depleted cells. Bottom: Images with over-enhanced green fluorescence intensities showing remaining RCC1-mACF signals are located in the MT-less region in the RCC1-knockdown spindle. **b** Live-cell images of RCC1 knockdown embryos suggesting that that the Type II configuration of nuclear-centrosome interaction (t=0) promotes asymmetrical spindle formation and unequal chromosome segregation (t=21) in RCC1-depleted embryos. Scale bars = 100 μm.

Supplementary References

1. Kaseda, K., McAinsh, A.D., and Cross, R.A. (2012). Dual pathway spindle assembly increases both the speed and the fidelity of mitosis. Biol Open *1*, 12-18. 10.1242/bio.2011012.
2. Kraeussling, M., Wagner, T.U., and Schartl, M. (2011). Highly asynchronous and asymmetric cleavage divisions accompany early transcriptional activity in pre-blastula medaka embryos. PLoS One *6*, e21741. 10.1371/journal.pone.0021741.
3. Iwamatsu, T., Shibata, Y., Hara, O., Yamashita, M., and Ikegami, S. (2002). Studies on fertilization in the teleost IV. Effects of aphidicolin and camptothecin on chromosome formation in fertilized medaka eggs. Dev Growth Differ *44*, 293-302. 10.1046/j.1440-169x.2002.00644.x.
4. Duro, J., and Nilsson, J. (2021). SAC during early cell divisions: Sacrificing fidelity over timely division, regulated differently across organisms: Chromosome alignment and segregation are left unsupervised from the onset of development until checkpoint activity is acquired, varying from species to species. Bioessays *43*, e2000174. 10.1002/bies.202000174.
5. Goshima, G., Nedelec, F., and Vale, R.D. (2005). Mechanisms for focusing mitotic spindle poles by minus end-directed motor proteins. J Cell Biol *171*, 229-240. 10.1083/jcb.200505107.
6. van Toorn, M., Gooch, A., Boerner, S., and Kiyomitsu, T. (2023). NuMA deficiency causes micronuclei via checkpoint-insensitive k-fiber minus-end detachment from mitotic spindle poles. Curr Biol *33*, 572-580 e572. 10.1016/j.cub.2022.12.017.
7. Wuhr, M., Tan, E.S., Parker, S.K., Detrich, H.W., 3rd, and Mitchison, T.J. (2010). A model for cleavage plane determination in early amphibian and fish embryos. Curr Biol *20*, 2040-2045. 10.1016/j.cub.2010.10.024.
8. Kimura, K., and Kimura, A. (2011). A novel mechanism of microtubule length-dependent force to pull centrosomes toward the cell center. Bioarchitecture *1*, 74-79. 10.4161/bioa.1.2.15549.
9. Laband, K., Le Borgne, R., Edwards, F., Stefanutti, M., Canman, J.C., Verbavatz, J.M., and Dumont, J. (2017). Chromosome segregation occurs by microtubule pushing in oocytes. Nat Commun *8*, 1499. 10.1038/s41467-017-01539-8.
10. Betterton, M.D., and McIntosh, J.R. (2013). Regulation of chromosome speeds in mitosis. Cell Mol Bioeng *6*, 418-430. 10.1007/s12195-013-0297-4.
11. Levy, D.L., and Heald, R. (2010). Nuclear size is regulated by importin alpha and Ntf2 in Xenopus. Cell *143*, 288-298. 10.1016/j.cell.2010.09.012.
12. Mukherjee, R.N., Chen, P., and Levy, D.L. (2016). Recent advances in understanding nuclear size and shape. Nucleus *7*, 167-186. 10.1080/19491034.2016.1162933.
