# Supplementary material for: Ran-GTP assembles a specialized spindle structure for accurate chromosome segregation in medaka early embryos
Source: Nat Commun. 2024 Feb 1;15:981. doi: 10.1038/s41467-024-45251-w (PMC10834446; doi:10.1038/s41467-024-45251-w)
Supplement: Supplementary file 3 — Description of Additional Supplementary Files [file 41467_2024_45251_MOESM3_ESM.pdf]

Description of Additional Supplementary Files

Supplementary Movie Legends:

Supplementary Movie 1: Related to Fig. 2a. Time-lapse movie of MIP images of a fertilized medaka embryo showing chromosomes (RCC1-mCh) and microtubules (GFP-α-tubulin) during early embryonic divisions.

Supplementary Movie 2: Related to Fig. 2c and Supplementary Fig. 2d. Time-lapse movie of MIP images (z2-4) of a medaka embryo showing cell cycle prolongation, cell migration, and abnormal divisions around late morula ~ early blastula stages.

Supplementary Movie 3: Related to Fig. 4i. Time-lapse movie of MIP images of a cold-treated medaka embryo showing MT regrowth after a temperature shift.

Supplementary Movie 4: Related to Fig. 6b and Supplementary Fig. 6c-d. Time-lapse movie of MIP images of an mCh-Ol-RanT27N-expressing embryo showing abnormal chromosome segregation during early embryonic divisions.

Supplementary Movie 5: Related to Fig. 7d. Time-lapse movie of MIP images of OsTIR1(F74G)-P2A-mCh-H2B expressing RCC1-mACF homozygous embryo showing abnormal chromosome segregation in response to the decrease of RCC1-mACF fluorescent signals
