# Supplementary material for: Ran-GTP assembles a specialized spindle structure for accurate chromosome segregation in medaka early embryos
Source: Nat Commun. 2024 Feb 1;15:981. doi: 10.1038/s41467-024-45251-w (PMC10834446; doi:10.1038/s41467-024-45251-w)
Supplement: Supplementary file 9 — Reporting Summary [file 41467_2024_45251_MOESM9_ESM.pdf]

Corresponding author(s): Tomomi Kiyomitsu

Last updated by author(s): Dec 27, 2023

Reporting Summary

Nature Portfolio wishes to improve the reproducibility of the work that we publish. This form provides structure for consistency and transparency in reporting. For further information on Nature Portfolio policies, see our Editorial Policies and the Editorial Policy Checklist.

Statistics

For all statistical analyses, confirm that the following items are present in the figure legend, table legend, main text, or Methods section.

n/a	Confirmed	
☐	☒	The exact sample size (*n*) for each experimental group/condition, given as a discrete number and unit of measurement
☐	☒	A statement on whether measurements were taken from distinct samples or whether the same sample was measured repeatedly
☐	☒	The statistical test(s) used AND whether they are one- or two-sided<br>*Only common tests should be described solely by name; describe more complex techniques in the Methods section.*
☒	☐	A description of all covariates tested
☐	☒	A description of any assumptions or corrections, such as tests of normality and adjustment for multiple comparisons
☐	☒	A full description of the statistical parameters including central tendency (e.g. means) or other basic estimates (e.g. regression coefficient) AND variation (e.g. standard deviation) or associated estimates of uncertainty (e.g. confidence intervals)
☐	☒	For null hypothesis testing, the test statistic (e.g. *F*, *t*, *r*) with confidence intervals, effect sizes, degrees of freedom and *P* value noted<br>*Give P values as exact values whenever suitable.*
☒	☐	For Bayesian analysis, information on the choice of priors and Markov chain Monte Carlo settings
☒	☐	For hierarchical and complex designs, identification of the appropriate level for tests and full reporting of outcomes
☒	☐	Estimates of effect sizes (e.g. Cohen's *d*, Pearson's *r*), indicating how they were calculated

Our web collection on statistics for biologists contains articles on many of the points above.

Software and code

Policy information about availability of computer code

Data collection	NIS-Elements (version 5.21.00)
Data analysis	NIS-Elements (version 5.20.00, and 5.40.00), Graphpad Prism 10 (version 10.0.2), Excel (version 16.61.1), Fiji (version 2.3.0/1.53q)

For manuscripts utilizing custom algorithms or software that are central to the research but not yet described in published literature, software must be made available to editors and reviewers. We strongly encourage code deposition in a community repository (e.g. GitHub). See the Nature Portfolio guidelines for submitting code & software for further information.

Data

Policy information about availability of data

All manuscripts must include a data availability statement. This statement should provide the following information, where applicable:

- Accession codes, unique identifiers, or web links for publicly available datasets
- A description of any restrictions on data availability
- For clinical datasets or third party data, please ensure that the statement adheres to our policy

All data supporting findings of this study are available in the paper and its Supplementary Information. Source data for each graph are provided as a separate Source Data Excel file.

Research involving human participants, their data, or biological material

Policy information about studies with human participants or human data. See also policy information about sex, gender (identity/presentation), and sexual orientation and race, ethnicity and racism.

Reporting on sex and gender	Not applicable
Reporting on race, ethnicity, or other socially relevant groupings	Not applicable
Population characteristics	Not applicable
Recruitment	Not applicable
Ethics oversight	Not applicable

Note that full information on the approval of the study protocol must also be provided in the manuscript.

Field-specific reporting

Please select the one below that is the best fit for your research. If you are not sure, read the appropriate sections before making your selection.

☒ Life sciences ☐ Behavioural & social sciences ☐ Ecological, evolutionary & environmental sciences

For a reference copy of the document with all sections, see nature.com/documents/nr-reporting-summary-flat.pdf

Life sciences study design

All studies must disclose on these points even when the disclosure is negative.

Sample size	Determined based on other relevant studies using embryos. Sample numbers are enough to do appropriate statistical tests.
Data exclusions	Embryos that had hatched normally after imaging were selectively used for quantification of cell cycle and spindles in Figs. 2b-c, 2g, 3b-e, 4c-d, 5b-c, and Supplementary Figs. 2b-c, and 3a-c to understand physiological intracellular dynamics.
Replication	All experiments were repeated at least 3 times, and similar results were obtained.
Randomization	The healthy fertilized eggs were used randomly.
Blinding	The investigators were not blinded for data collection and quantification because chromosome segregation or other phenotypes are associated with other detectable factors such as decrease of fluorescence level of RCC1-mACF or specific localization of mCh-Ol-RanT27N.

Reporting for specific materials, systems and methods

We require information from authors about some types of materials, experimental systems and methods used in many studies. Here, indicate whether each material, system or method listed is relevant to your study. If you are not sure if a list item applies to your research, read the appropriate section before selecting a response.

Materials & experimental systems

n/a	Involved in the study	
☐	☒	Antibodies
☒	☐	Eukaryotic cell lines
☒	☐	Palaeontology and archaeology
☐	☒	Animals and other organisms
☒	☐	Clinical data
☒	☐	Dual use research of concern
☒	☐	Plants

Methods

n/a	Involved in the study	
☒	☐	ChIP-seq
☒	☐	Flow cytometry
☒	☐	MRI-based neuroimaging

Antibodies

Antibodies used	The primary antibodies: anti-α-tubulin mouse monoclonal antibody (Sigma-Aldrich, T6199, 1:200 dilution) and anti-gamma-tubulin mouse monoclonal antibody (Sigma-Aldrich, T6557, 1:200 dilution). Secondary antibodies: goat anti-mouse IgG Alexa Fluor Plus 555

	(Invitrogen, A32727, 1:1000 dilution) or goat anti-mouse IgG Alexa Fluor Plus 647 (Invitrogen, A32728, 1:1000 dilution)
Validation	These antibodies are commonly used for immuno-fluorescence in several research papers including Inoue et al., Nature Communications 2017.

Animals and other research organisms

Policy information about studies involving animals; ARRIVE guidelines recommended for reporting animal research, and Sex and Gender in Research

Laboratory animals	Medaka OK-Cab strain was used. Fertilized eggs were collected from 2-9 month old pairs.
Wild animals	Not applicable
Reporting on sex	Not applicable
Field-collected samples	Not applicable
Ethics oversight	The Animal Care and Use Committee at Okinawa Institute of Science and Technology Graduate University (OIST)

Note that full information on the approval of the study protocol must also be provided in the manuscript.

Plants

Seed stocks	Not applicable
Novel plant genotypes	Not applicable
Authentication	Not applicable
